# Pentraxin-3 regulates the inflammatory activity of macrophages

**DOI:** 10.1016/j.bbrep.2016.01.009

**Published:** 2016-01-14

**Authors:** Aya Shiraki, Norihiko Kotooka, Hiroshi Komoda, Tetsuaki Hirase, Jun-ichi Oyama, Koichi Node

**Affiliations:** a Department of Cardiovascular Medicine, Faculty of Medicine, Saga University, 5-1-1 Nabeshima, Saga, Japan; b National Cerebral and Cardiovascular Center Research Institute, 5-7-1, Fujishirodai, Suita, Osaka, Japan

**Keywords:** Pentraxins, PTX3, Macrophages, Inflammation

## Abstract

**Background and aims:**

Pentraxin-3 (PTX3) reportedly has protective roles in atherosclerosis and myocardial infarction, and is a useful biomarker of vascular inflammation. However, the detailed functions of PTX3 in inflammation are yet to be elucidated. This study aimed to investigate the function of PTX3 in macrophages.

**Methods:**

PMA-treated THP-1 cell line (THP-1 macrophage) and monocyte-derived human primary macrophages were treated with recombinant PTX3. Cytokine and chemokine levels in the THP-1 culture medium were measured as well as monocyte chemoattractant protein (MCP-1) concentrations in the Raw 264.7 cell culture medium. PTX3-silenced apoptotic macrophages (THP-1 cell line) were generated to investigate the roles of PTX3 in phagocytosis.

**Results:**

In the presence of PTX3, macrophage interleukin-1β (IL-1β), tumor necrosis factor-alpha (TNF-α) and MCP-1 levels were reduced significantly (−39%, *P*=0.007; −21%, *P*=0.008; and −67%, *P*=0.0003, respectively), whilst activated transforming growth factor-β (TGF−β) was detected in the THP-1 macrophages (*P*=0.0004). Additionally, PTX3 induced Akt phosphorylation and reduced nuclear factor-kappa B (NF-κB) activation by 35% (*P*=0.002), which was induced by TNF-α in THP-1 macrophages. Furthermore, silencing of PTX3 in apoptotic cells resulted in increased macrophage binding, elevated expression rate of HLA-DR (+30%, *P*=0.015) and CD86 (+204%, *P*=0.004) positive cells, and induction of IL-1β (+36%, *P*=0.024) production. Conversely, adding recombinant PTX3 to macrophages reduced CD86 and HLA-DR expression in a dose-dependent manner.

**Conclusions:**

We identified PTX3 as a novel regulator of macrophage activity, and this function suggests that PTX3 acts to resolve inflammation.

## Highlights

• PTX3 reduces pro-inflammatory cytokines from macrophages and induced active TGFβ.
• PTX3 upregulates Akt phosphorylation in macrophages.
• PTX3 reduces TNFα induced NFκ-B activation.
• Silencing PTX3 of apoptotic cells evokes phagocytic activity of macrophages.

## Graphical abstract

**Figure:**
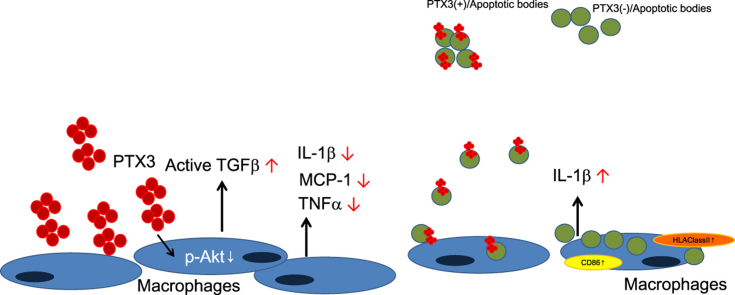

## 1 Introduction

Inflammation, resolution of inflammatory processes and tissue repair, are pivotal factors in many diseases. When inflammation occurs, many cytokines and mediators, including pentraxins, are produced as needed for the immune response. During healing, positive processes are evoked to resolve the inflammation and activate multiple inhibitory pathways, including local transforming growth factor-beta (TGF-β) activation, recruitment of suppressive monocytes and upregulation of M2 macrophages and regulatory T cells, which can produce and secrete inhibitory mediators such as TGF-β and interleukin (IL)-10 [1].

Pentraxins are a superfamily of pattern recognition proteins that constitute the prototypic components of the humoral arm of the innate immune system. C-reactive protein (CRP) and serum amyloid P component (SAP) are short pentraxins produced by the liver. In contrast, pentraxin-3 (PTX3) was the first long pentraxin identified as an IL-1-inducible protein in endothelial cells and a tumor necrosis factor (TNF)-stimulated protein in fibroblasts [2], [3].

PTX3 assembles into a four protein tetramer, with two links together forming an octameric structure [4]. PTX3 recognizes pathogen-associated molecular patterns. Moreover, it interacts with C1q, ficolin-1, ficolin-2 and mannose-binding lectin, all of which are recognition molecules in the classical and lectin complement pathways [3]. Furthermore, PTX3 has been found to have significant interaction with fibroblast growth factor (FGF)-2 [5], Fcγ receptor [6] and P-selectin [7].

PTX3-deficient mice exhibit exacerbated myocardial damage following coronary artery ligation and reperfusion associated with greater no-reflow areas, increased neutrophil infiltration, decreased numbers of capillaries and increased numbers of apoptotic cardiomyocytes [8]. Moreover, double-knockout mice, lacking PTX3 and apolipoprotein E, exhibit increased atherosclerosis and macrophage accumulation in atherosclerotic lesions compared with that observed in apolipoprotein E only knockout mice, suggesting that PTX3 possesses a cardiovascular protective function [9]. Furthermore, PTX3 is also reported to be expressed in atheromas [10], [11]. However, the detailed mechanisms underlying these functions of PTX3 remain to be elucidated.

In clinical studies, the usefulness of PTX3 as a biomarker has emerged in many diseases, including unstable angina pectoris [12], acute coronary syndrome [13], chronic heart failure [14], heart failure with a normal ejection fraction [15], and Takayasu arteritis [16]. Upregulation of PTX3 in the serum is considered to be an indicator of vascular inflammation [17]; however, little evidence is available regarding the role and results of PTX3 upregulation.

Although some studies have been carried out to investigate the relationship between PTX3 and macrophages, the function of PTX3 in macrophages has not been studied to date. In the present study, we investigated the influence of PTX3 in macrophages in vitro, and found that it regulated inflammatory activities of these cells.

## 2 Materials and methods

### 2.1 Ethics statement

The research met all applicable standards for the ethics of experimentation and was approved by the Ethics Committee of the Faculty of Medicine of Saga University (No. 26-56). Participants provided written informed consent prior to the experiment.

### 2.2 Cell culture and materials

Cells were cultured at 37 °C under 5% CO_2_. The human THP-1 cell line was purchased from Riken Bioresource Center (RCB1189). THP-1 cells were cultured in RPMI medium with 10% fetal bovine serum (FBS). THP-1 macrophages were generated by incubating the cells with 100 nmol/L of phorbol-myristate-acetate (PMA) for 24 h. Raw 264.7 cells were purchased from ATCC and cultured in DMEM with 2 mM Glutamine and 10% Foetal Bovine Serum (FBS). Recombinant PTX3 (rPTX3) was provided by Perseus Proteomics Inc. (Tokyo, Japan). It was made by the procedure that was written in the article [12]. Human umbilical vein endothelial cells (HUVECs) and specific medium for HUVEC culture were obtained from Lonza (Basel, Swiss). Experiments using HUVECs were conducted with cells between passage numbers three and five (P3–P5). Human monocyte-derived macrophages were generated from healthy volunteers' blood.

Peripheral blood mononuclear cells (PBMC) were separated using Ficoll-Paque PLUS solution and lymphocytes were washed away after monocyte adhesion on tissue culture-treated culture plates. Cells were cultured with 20 ng/mL of granulocyte macrophage-colony stimulating factor (GM-CSF, Pepro Tech, Rocky Hill, NJ) in RPMI medium containing 10% FBS and were incubated for 6 days prior to assaying. TGF-β neutralizing antibody was obtained from R&D Systems (Minneapolis, MN. Ab-100-NA rabbit polyclonal, 10 µg/mL).

### 2.3 Western blot analysis

THP-1 macrophages were lysed with RIPA buffer containing sodium fluoride (NaF), trypsin inhibitor, leupeptin, β-glycerophosphate and orthovanadate. Lysates of samples were resolved on SDS-PAGE according to standard protocol. The used protein amount was 40 μg/lane. After being transferred to membranes, samples were immunoblotted with primary antibodies (see below) followed by secondary antibodies conjugated to horseradish peroxidase (NA934V anti-rabbit IgG, NA931V anti-mouse IgG, GE Healthcare, Buckinghamshire, UK). Bands were revealed using ECL Plus Western Blotting Detection Reagents or ECL Advance Western Blotting Detection Reagents (GE Healthcare, Buckinghamshire, UK). The band density was quantified using Image J software [18]. The following primary antibodies were used: anti-p-Akt (#9271 rabbit polyclonal, 1:50), anti-Akt (#9272 rabbit polyclonal, 1:1000), which were purchased from Cell Signaling (Danvers, MA). Actin (I-19): (sc-1616-R, rabbit polyclonal, 1:1000) was from Santa Cruz Biotech (Santa Crus, CA). All western blots were performed at least three times.

### 2.4 Cytokine measurements

TNF-α, IL-1β, monocyte chemoattractant protein-1 (MCP-1), active TGF-β and IL-10 were measured in the culture medium using the Luminex 100 system (Luminex Corporation, Austin, TX). Anti-human Procarta Cytokine Plex kits (PN-PC1004) were purchased from Veritas (Tokyo, Japan). Some data for murine MCP-1 and human IL-1β were obtained from enzyme-linked immunosorbent assays (ELISA) using commercially available kits from eBioscience (San Diego, CA). To measure active TGF-β, samples were not put through an acidifying and neutralizing procedure.

### 2.5 NF-κB activation

To study NF-κB activation, HUVECs were transiently transfected with the pGL4.32[luc2P/NFκB-RE/Hygro] vector, containing five copies of the NF-κB response elements, to drive transcription of the luciferase reporter gene luc2P. This NF-κB-RE reporter plasmid was transfected by electroporation, together with the phRL-CMV plasmid that is renilla luciferase control reporter vector, into HUVECs using a Gene Pulser Xcell (square wave, 100 V, 20 ms, BioRad, Hercules, CA). The cells were cultured overnight and starved in RPMI-1640 medium containing 0.1% FBS, heparin (500 µ/L) and endothelial cell growth factor (ECGF, 2 mg/L) for 1 h and pre-incubated with either rPTX3 (100 ng/mL) or vehicle for 30 min. The cells were then stimulated with TNF-α (5 ng/mL) for 6 h. Luciferase activity was then measured with the Dual-Luciferase Reporter Assay System (Promega, Madison, WI) and expression levels of luc2P (Photinus pyralis) was standardized by the renilla luciferase activity.

### 2.6 Silencing PTX3 and inducing apoptosis in THP-1 cells

THP-1 cells were seeded at 80,000 cells/well in 96-well plates and incubated with 100 nM PMA overnight. Wells were rinsed twice with RPMI-1640 containing l-glutamine and 10% FBS and replaced with 100 μL medium. The siRNA for PTX3 and control siRNA were purchased from Santa Cruz Biotechnology (sc-39187 and sc-37007, respectively, Dallas, Texas). siRNA (13.3 pmol/well) was diluted in 25 μL Opti-Mem medium. Lipofectamine® (1.66 µL) RNAiMAX Transfection Reagent, purchased from Life Technologies (Carlsbad, California), was diluted in 25 μL Opti-Mem medium and these two dilutions were mixed gently and incubated for 10–20 min at room temperature. This 50-μL mixture was then added to THP-1 macrophages generated in 96-well plates. The cells were incubated for 24 h at 37 °C in a CO_2_ incubator. The knockdown efficacy was 85% when stimulated by lipopolysaccharide (LPS) (Supplementary data), which was determined by a PTX3 ELISA purchased from Perseus Proteomics (Tokyo, Japan).

### 2.7 Apoptotic cell generation and binding assay

THP-1 macrophages were treated with siRNA to reduce levels of PTX3 expression. PTX3-low and PTX3-normal macrophages were then exposed to UV irradiation (302 nm, 0.07 w/cm^2^) for 10 min to induce apoptosis. These apoptotic macrophages were immediately stained with Cell Tracker Blue (Invitrogen) and added to a monolayer of THP-1 macrophages in 96-well plates and incubated for 24 h. Non-adherent apoptotic macrophages were washed twice with RPMI medium and photos were captured by a FSX100 microscope (Olympus, Japan).

### 2.8 Immunocytochemistry

THP-1 cells were fixed with 10% neutral buffered formalin fixative and rinsed with phosphate buffered saline (PBS). Non-permeabilized cells were blocked with PBS containing 3% bovine serum albumin (BSA). Cells were incubated with primary antibodies to HLA-DR (12-9956, mouse monoclonal, 1:20) and CD86 (12-0869 mouse monoclonal,1:5) (eBioscience, San Diego, CA). Cells were then rinsed and incubated with secondary antibody (A11061 rabbit polyclonal anti-mouse IgG, Alexa Fluor 568, Invitrogen) at room temperature for 30 min. Hoechst 33342 (Invitrogen) was used for nuclear staining and images were captured by a FSX100 microscope. Positive cells were analyzed with Image-J software.

### 2.9 Flow cytometry

Human monocyte-derived macrophages were washed twice with PBS and incubated in PBS/EDTA for 10 min prior to collection. HLA-DR-FITC antibody (eBioscience, 11-9956 mouse monoclonal, 2.5 µL/test) and CD86-PE antibody (eBioscience, 12-0869 mouse monoclonal, 2.5 µL/test) were incubated with the samples for 30 min. Those samples were washed twice with 1%FBS/PBS. The antibodies of isotype controls were also used. FITC and PE levels were measured by the MACSQuant analyzer (Miltenyi Biotec. Germany).

### 2.10 Statistical analysis

Data are expressed as the means±standard deviation (SD). Student's *t* test was used to determine differences between two population averages. Data comparisons between groups were carried out using one-way analysis of variance (ANOVA) with a Bonferroni post-hoc test. A *P* value less than 0.05 was deemed to be statistically significant. All statistical analyses were performed using the Statistical Package for the Social Sciences (SPSS 16.0 Japanese edition for Windows, SPSS Inc. Tokyo, Japan).

## 3 Results

### 3.1 rPTX3 reduced pro-inflammatory cytokines and induced active TGF-β in THP-1 cells

To elucidate the role of protein PTX3 in macrophages, one of the main players in atherosclerosis, human rPTX3 was used. Following serum depletion for 1 h, THP-1 macrophages were treated with 10 ng/mL of rPTX3 and the cells were incubated for 24 h. We used a PTX3 concentration of 10 ng/mL because this is the reported concentration level in human blood when inflammation occurs, such as during acute myocardial infarction [19].

**Fig. 1:**
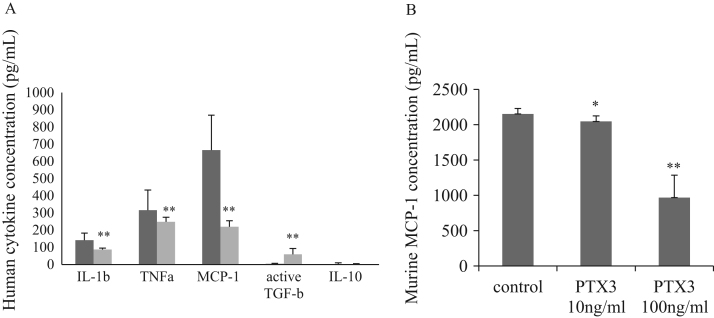
rPTX3 reduced inflammatory cytokines and induced active TGF-β (A) THP-1 macrophage-conditioned culture medium was assessed for cytokines using the Luminex system. The culture medium was serum-depleted and incubated with THP-1 cells for 24 h with (grey columns) or without 10 ng/mL rPTX3 (black columns), (means±SD, *n*=8; **p<0.01); (B) rPTX3 reduced MCP-1 production by murine Raw 264.7 macrophage cells, activated by 20 nM of PMA and cultured with or without human rPTX3 for 24 h. The cultured medium was collected and measured using ELISA (means±SD, *n*=8; *p<0.05, **p<0.001). PMA: phorbol-myristate-acetate.

The Luminex assay showed that rPTX3 reduced the pro-inflammatory cytokines TNF-α, IL-1β and MCP-1 (Fig. 1A). To assess the quantity of originally active TGF-β, acidifying and neutralizing procedures were omitted before the Luminex assay. Consequently, rPTX3 was able to induce active TGF-β. Interestingly, IL-10 was not detectable in the cultured medium.

To clarify whether rPTX3 acted in the same way in different types of macrophages, we activated murine Raw 264.7 cells with 20 mol/L of PMA and treated them with 10 ng/mL or 100 ng/mL of rPTX3. We found that exposure to rPTX3 reduced the concentration of MCP-1 in the culture medium of Raw 264.7 cells (Fig. 1B). The murine MCP-1 ELISA assay was chosen because larger differences in MCP-1 levels by human THP-1 macrophages were detected using the Luminex assay.

### 3.2 rPTX3 induced phosphorylation of Akt in THP-1 cells

**Fig. 2:**
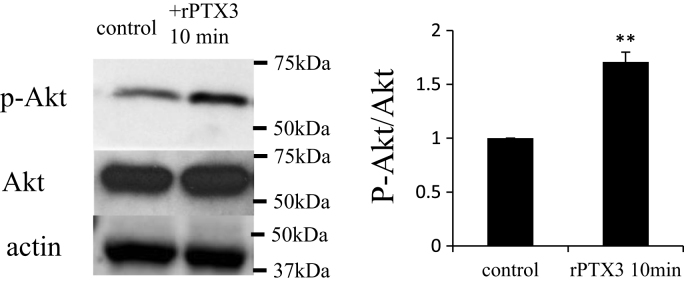
rPTX3 modulated intracellular signaling. Western blotting displayed increased phosphorylation of Akt in THP-1 macrophages. The graphs show the densitometry value analysis of western blots. The western blots were performed at least three times (**p<0.001).

We investigated intracellular signaling in THP-1 macrophages to detect any cellular responses following PTX3 treatment. Surprisingly, rPTX3 treatment of THP-1 macrophages obviously induced phosphorylation of Akt in 10 min (Fig. 2).

### 3.3 rPTX3 reduced TNF-α-induced NF-κB activation

**Fig. 3:**
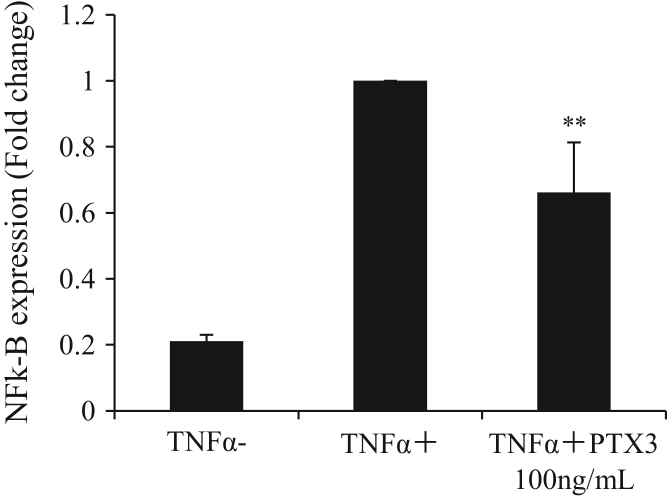
rPTX3 reduced TNFα-induced NFκB activation. HUVECs were transfected with pGL4.32[luc2P/NFκB-RE/Hygro] reporter vector and TNF-α was added. Pre-treatment with rPTX3 reduced NF-κB activation (means±SD, *n*=5; **p<0.001 vs. TNF-α(+)).

Next, we determined whether NF-κB activation was involved in the rPTX3-mediation process. A luciferase reporter assay was performed by transfecting pGL4.32[luc2P/NFκB-RE/Hygro] vectors into HUVECs. The activation level of NF-κB induced by TNF-α was reduced by rPTX3 (−34%, *P*=0.002) (Fig. 3).

### 3.4 Silencing PTX3 enhanced inflammatory responses in THP-1 macrophages

**Fig. 4:**
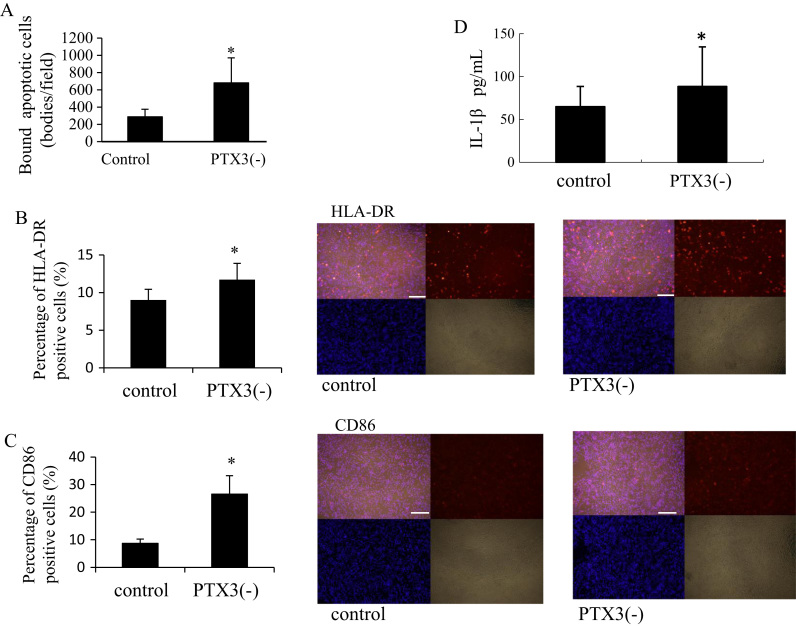
Silencing PTX3 in apoptotic cells raised the affinity for macrophages and attenuated inflammatory responses. (A) Binding of apoptotic bodies on phagocytes was elevated in PTX3(-) THP-1 cells (means±SD, *n*=8, *p<0.005). (B,C) Immunocytochemistry of phagocytic THP-1 macrophages. HLA-DR and CD86 were immunostained with Alexa Fluoro 568 secondary antibody (red). Hoechst 33342 (blue) was used for nuclear staining. Photos: upper left, merged image; upper right, HLA-DR or CD86; lower left, Hoechest 33342; lower right, phase-contrast image. Bar, 0.3 mm. Positive cells were counted and normalized to the number of the cells present to generate the percentage using Image-J (means±SD, *n*=8; *p<0.05). (D) IL-1β concentration in cultured medium (means±SD, *n*=8; *p<0.05).

To further elucidate the effects of PTX3, we developed an assay to determine how the knockdown of PTX3 expression in apoptotic cells might affect the binding and engulfment ability of macrophages and the inflammatory response. PTX3-low macrophages were generated by siRNA and the cells were then treated with UV to induce apoptosis. As a result, PTX3-low apoptotic cells bound to macrophages at more than twice the frequency of PTX3-normal apoptotic cells (Fig. 4A). The macrophages that engulfed apoptotic bodies were analyzed by staining for HLA-DR and CD86. The frequencies of HLA-DR- and CD86-positive cells were significantly elevated in macrophages that engulfed PTX3-low apoptotic bodies (Fig. 4B and C). IL-1β was also upregulated in the culture medium in the PTX3-low group (Fig. 4D).

### 3.5 rPTX3 regulated expression of HLA-DR and CD86 in macrophages

**Fig. 5:**
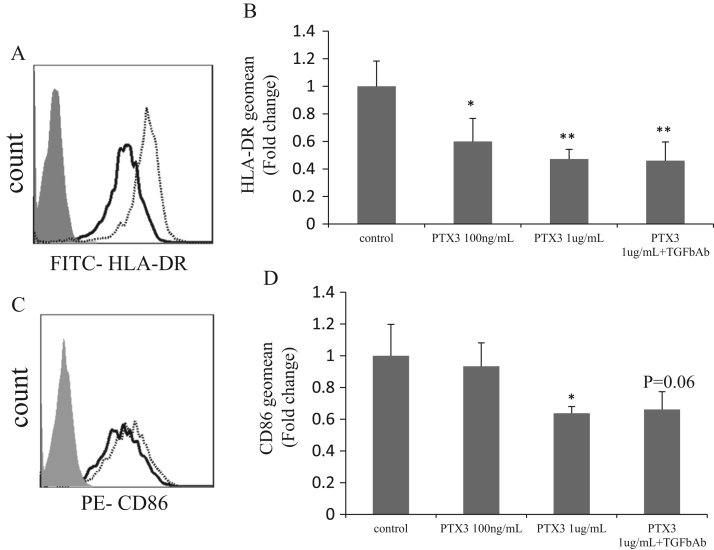
rPTX3 reduced HLA-DR and CD86 expression in human monocyte-derived macrophages. Macrophages were generated from monocytes cultured with 20 ng/mL GM-CSF for 6 days and incubated with rPTX3 for another 24 h. (A,C) Representative data of flow cytometric histograms of human macrophages. Filled histograms, isotype IgG; dotted line, control; solid line, PTX3 1 µg/mL. (B and D) Geometric means of HLA-DR and CD86 on macrophages (means±SD, *n*=3–8; **p<0.005,*p<0.05).

We attempted to clarify the mechanism by which PTX3-silencing upregulated HLA-DR and CD86. We therefore investigated if rPTX3 reduced HLA-DR and CD86 expression in macrophages. To obtain a homogeneous population of macrophages and to ensure rPTX3 could work effectively on primary macrophages, human monocyte-derived macrophages were generated. On the 6th day, human monocyte-derived macrophages were cultured with 1 µg/mL rPTX3 for 24 h. Expression levels of HLA-DR and CD86 were significantly reduced by rPTX3 (Fig. 5). Furthermore, treatment with TGF-β neutralizing antibody did not reverse this effect.

## 4 Discussion

This report demonstrates that PTX3 promoted activated TGF-β and induced anti-inflammatory responses in macrophages. This anti-inflammatory property is a novel function of PTX3 that may play an important role in inflammation and the subsequent healing process. In this report, human recombinant PTX3 was also treated in RAW 264.7 cells, which were mouse origin, and it worked. Originally, the protein PTX3 was well conserved through species and the identity of protein PTX3 between human and mouse was 82%.

Here, we investigated the function of PTX3 in macrophages. Surprisingly, the levels of pro-inflammatory cytokines released from macrophages decreased in the presence of rPTX3. Moreover, active TGF-β was detected in serum-free culture medium. This result suggests that PTX3 acts to suppress the activity of macrophages at inflamed sites. Recently, it was reported that 50% of PTX3-positive macrophages in coronary atherosclerosis were M2 type [10]. Moreover, Hua et al. reported that heavy chain–hyaluronic acid complex (HC–HA), purified from the human amniotic membrane, contains PTX3, induces TGF-β1 and reduces TNF-α in RAW264.7 cells [20]. They also found that the HC–HA-PTX3 complex is produced by amniotic membrane cells and propose that it has a protective role in fetal development. Interestingly, amniotic membrane is used in various operative situations, such as amniotic membrane grafting in ophthalmologic procedures and in the dressing of burns, because it has anti-inflammatory, anti-scarring and anti-angiogenic actions. Our finding showed that PTX3 itself suppressed macrophage pro-inflammatory activity, which may be one explanation of the protective role of the amniotic membrane during fetal development and wound healing.

Activation of TGF-β is promoted by a wide range of factors, including proteases, physiochemical mediators and binding of other proteins, such as thrombosponsin-1 and integrins [21]. In this study, we detected active TGF-β within macrophage culture medium after adding rPTX3. Unfortunately, it remains unclear how TGF-β was activated by PTX3 under these conditions. The upregulation of TGF-β was not responsible for the reduction of HLA-DR and CD86 expression by human macrophages. Thus, the signaling pathway used by rPTX3 in macrophages remains to be identified.

Our observation that PTX3 modulated intracellular signaling by p-Akt is novel. However, we did not do further experiments using inhibitors of Akt such as wortmannin and LY294002, because macrophages and HUVECs did not survive with those inhibitors when incubation periods were over 12 h, that was necessary for the expressions of property of rPTX3. The roles of Akt activation remained to be elucidated.

Other researchers have reported the existence of cellular receptors for PTX3 in macrophages [22]. Although PTX3 has also been reported to interact with the Fcγ receptor, it remains to be established whether other PTX3 receptors exist [4].

Some studies have reported that PTX3 can act directly on cells. For example, PTX3 inhibits phagocytosis of late apoptotic cells by macrophages [23] and dendritic cells (DCs) [24], although the underlying mechanisms are not clear. In view of these findings, our results showed that silencing of PTX3 caused increased binding of apoptotic bodies on macrophages, indicating PTX3 also has an inhibitory effect on phagocytosis.

In our experiments, rPTX3 decreased membrane expression of CD86 and HLA-DR/HLA-class II in human-derived macrophages. Baruah et al., have shown that PTX3 influences membrane expression of CD86 and MHC class I and II on DCs when activated by LPS [25]. We believe a similar phenomenon has been observed here with macrophages, although the decreased expression of CD86 and HLA-DR could be observed without LPS stimulation. To elucidate the possible involvement of TGF-β activation, TGF-β-neutralizing antibody was used. The regulation of cell surface markers by PTX3 seemed to be independent of TGF-β because they were not affected by TGF-β blockage.

During acute inflammation, the blood concentration of PTX3 rapidly elevates. For instance, plasma PTX3 levels rise during early acute myocardial infarction [26]. This is most likely due to release of activated neutrophils. The upregulation of PTX3 can be attributed to transcriptional factors such as NF-κB in monocytes, macrophages and endothelial cells.

Our findings suggest that PTX3 attenuates and regulates excessive inflammatory activity in macrophages and promotes the healing process. We propose that PTX3 could be a new treatment for acute inflammation, especially in macrophage-related wounds, such as following myocardial infarction, in atherosclerotic lesions, and in systemic inflammation in which macrophages participate.

PTX3 attenuated inflammatory activity in cultured macrophages. These functional results suggest that PTX3 may participate in the regulation of inflammation.

## Footnotes

Appendix A Supplementary data associated with this article can be found in the online version at doi:10.1016/j.bbrep.2016.01.009.
