# Supplementary material for: Pentraxin-3 regulates the inflammatory activity of macrophages
Source: Biochem Biophys Rep. 2016 Jan 14;5:290–5. doi: 10.1016/j.bbrep.2016.01.009 (PMC5600337; doi:10.1016/j.bbrep.2016.01.009)
Supplement: Supplementary file 1 — Supplementary material [file mmc1.zip › mmc1.pptx]

## Slide 1
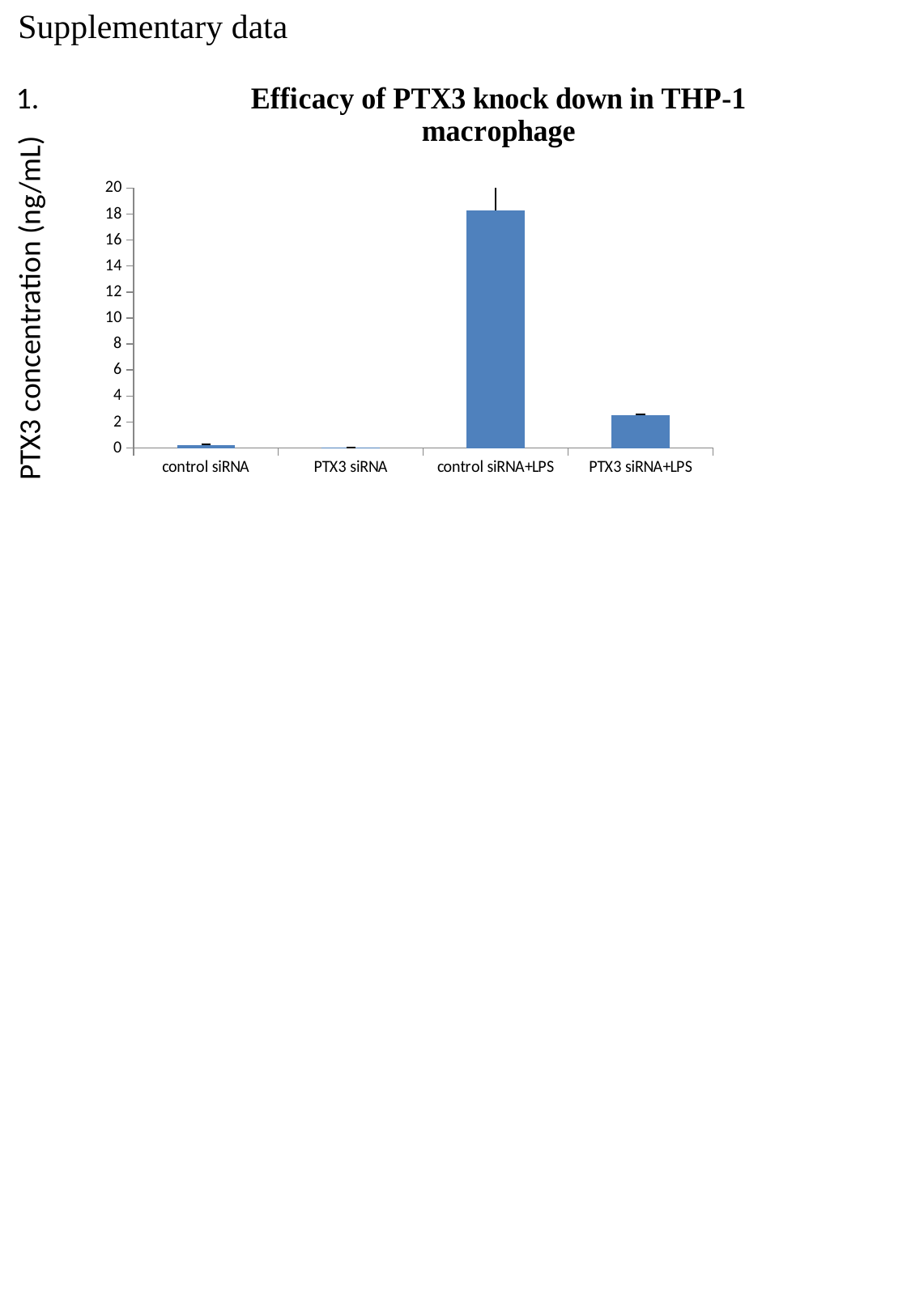

# Supplementary data
1.
### Chart: Efficacy of PTX3 knock down in THP-1 macrophage
| Category | PTX3 concentration ng/ml |
|---|---|
| control siRNA | 0.23275 |
| PTX3 siRNA | 0.034 |
| control siRNA+LPS | 18.2675 |
| PTX3 siRNA+LPS | 2.5 |PTX3 concentration (ng/mL)

## Slide 2
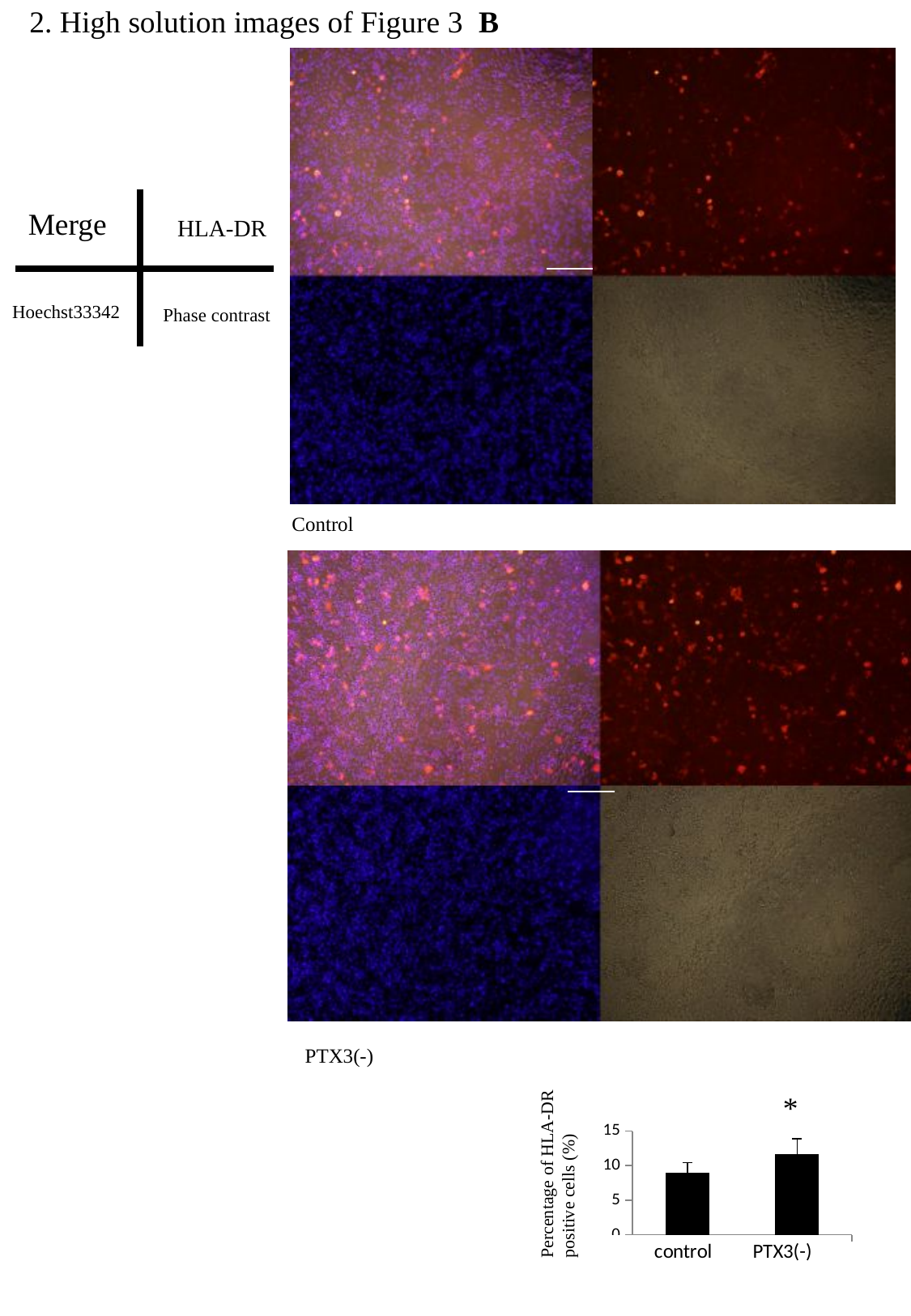

2. High solution images of Figure 3 B
Merge
HLA-DR
Hoechst33342
Phase contrast
Control
PTX3(-)
*
### Chart
| Category | |
|---|---|Percentage of HLA-DR
positive cells (%)

## Slide 3
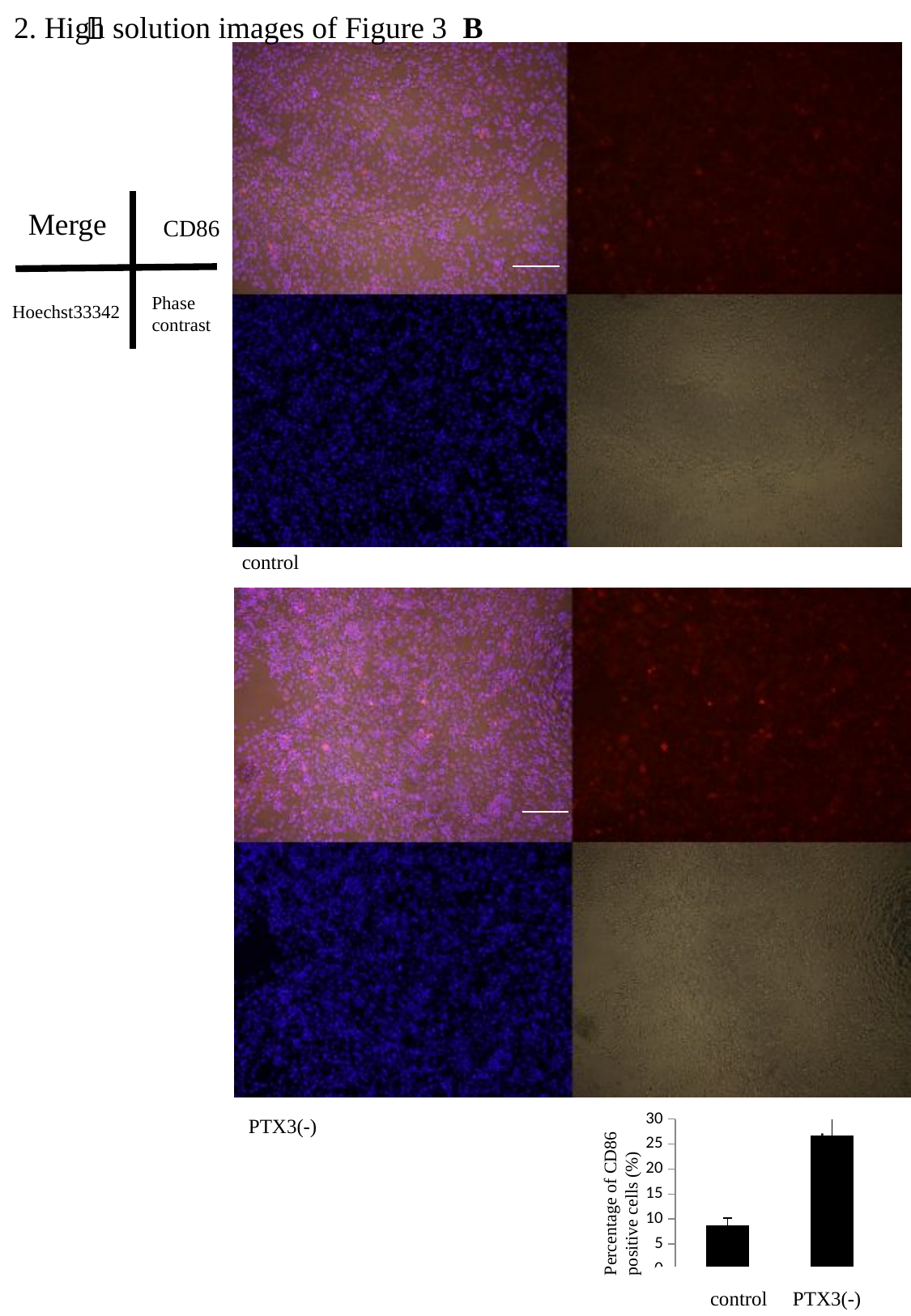

2. High solution images of Figure 3 B
Ｃ
Merge
CD86
Phase
contrast
Hoechst33342
control
### Chart
| Category | |
|---|---|PTX3(-)
*
Percentage of CD86
positive cells (%)
control PTX3(-)
